# Tumor Size Is an Independent Prognostic Factor for Stage I Ovarian Clear Cell Carcinoma: A Large Retrospective Cohort Study of 1,000 Patients

**DOI:** 10.3389/fonc.2022.862944

**Published:** 2022-05-16

**Authors:** Liangcai Wu, Shuo Shi, Hong Sun, Haiyan Zhang

**Affiliations:** Obstetrics and Gynecology Hospital, Fudan University, Shanghai, China

**Keywords:** ovarian clear cell carcinoma (OCCC), tumor size, prognosis factor, early stage, propensity score matching, inverse probability weighting analysis

## Abstract

**Objective:**

The aim of this study was to investigate the prognostic value and stratification cutoff point for tumor size in stage I ovarian clear cell carcinoma (OCCC).

**Methods:**

This was a retrospective cohort study using the Surveillance, Epidemiology, and End Results database (version: SEER 8.3.9). Patients diagnosed with stage I OCCC from 1988 to 2018 were included for further analysis. X-Tile software was used to identify the potential cutoff point for tumor size. Stratification analysis, propensity score matching, and inverse probability weighting analysis were used to balance the potential confounding factors.

**Results:**

A total of 1,000 stage I OCCC patients were included. Of these 1,000 patients, median follow-up was 106 months (95% confidence interval [CI]: 89–112 months). Multivariate analysis showed that tumor size, age at diagnosis, and stage IC were significantly associated with stage I OCCC patients. Eight centimeters is a promising cutoff point that can divide stage I OCCC patients into a good or a poor prognosis group. After controlling potential confounding factors with propensity score matching and inverse probability weighting, we demonstrated that stage I OCCC patients with tumor size ≤ 8 cm enjoyed a significantly better 5-year overall survival (OS, 89.8% vs. 81%, *p* < 0.0001). Tumor size ≤ 8 cm was an independent prognostic factor of stage I OCCC patients (hazard ratio [HR] 0.5608, 95% CI: 0.4126–0.7622, *p* = 0.0002).

**Conclusions:**

Tumor size is an independent prognostic factor for stage I OCCC, and 8 cm is a promising cutoff point for tumor size for risk stratification. However, using tumor size in the stratification management of stage I OCCC patients warrants further investigation.

## Introduction

Ovarian clear cell carcinoma (OCCC) is a relatively rare (incidence: 3%–10%) but distinct histological type of epithelial ovarian cancer (1, 2). Unlike high-grade serous adenocarcinoma (HGSOC), stage I OCCC accounts for 56.3%–65.5% (3). Moreover, many OCCC cases were diagnosed during minimally invasive surgery for ovarian cyst excision (4). It was reported that minimally invasive surgery was a promising therapeutic option in early-stage ovarian epithelial cancer (5–7). Endometriosis was regarded as a precursor of OCCC, and one single-center retrospective study revealed that OCCC with concurrent endometriosis accounts for 45% of all OCCC cases (8). Moreover, clear cell carcinoma was frequently mixed with other histological types of cancer (9) and was associated with a poorer prognosis (10).

Even though OCCC is diagnosed at an early stage and in younger patients, the prognosis of OCCC seems unfavorable (3, 11, 12). A previous large retrospective cohort study showed that OCCC patients have a significantly worse 5-year overall survival compared with patients with HGSOC in every sub-FIGO stage analysis (3). It seems that prognosis of early-stage OCCC is heterogeneous (11, 12). Many stage IA CCC patients have quite a favorable disease-free survival rate, while a subset of OCCC patients progress quickly and experience recurrence (13).

Tumor size is considered a tumor burden parameter, and this parameter is used to evaluate clinical response or prognosis (14, 15). Tumor size also reflects the complexity of the tumor ecosystem (16). During tumor growth, intra-tumoral heterogeneity is dramatically increased, with cancer stem cells and a number of driver mutations (17–19). However, the prognostic effect of tumor size in early-stage OCCC has not been well explored. Thus, we asked whether tumor size in stage I OCCC can predict prognosis. In the current study, we used the largest public database, the Surveillance, Epidemiology, and End Results (SEER) database, to evaluate the prognostic value of primary tumor size in patients with OCCC.

## Method and Materials

We conducted this retrospective cohort study according to the STROBE statement and used the high-quality open access database, the SEER database (version 8.3.9).

### Data Extraction and Screening

**Figure 1:**
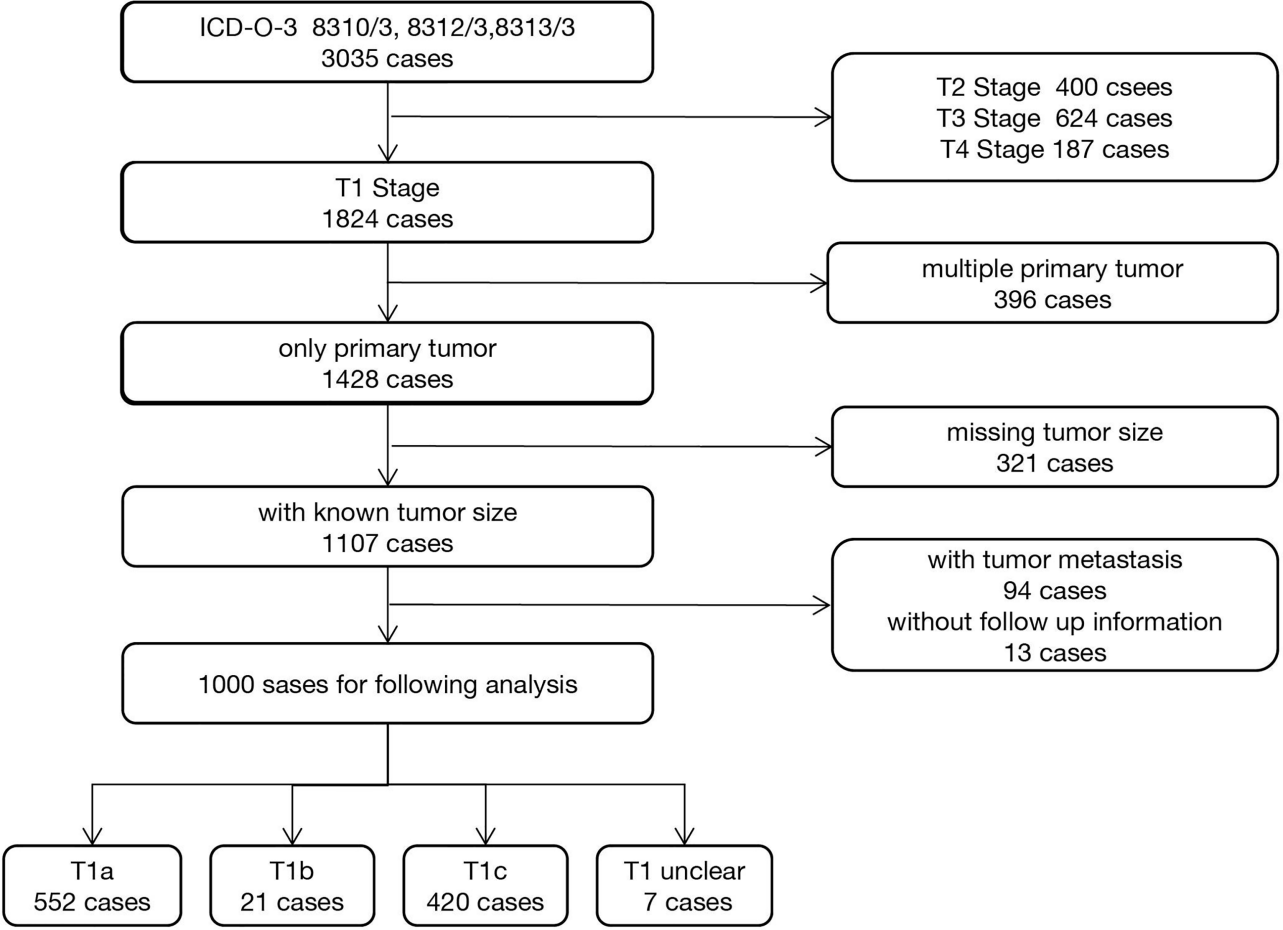
Workflow of 1,000 ovarian clear cell carcinoma patients’ selection from the Surveillance, Epidemiology, and End Results (SEER) database.

After registration and approval by the SEER team, we downloaded the SEER*Stat 8.3.9 software and established a local OCCC database by extracting the raw clinical information and pathological information from 1998 to 2018. According to the ICD-O-3 coding system, we identified malignant OCCC using the following codes: 8310-3, 8312-3, and 8313-3. Clinical information, such as race, year of diagnosis, sequence of diagnosis, age, survival (months), and vital status, was extracted. Tumor characteristics, such as TNM stage, histological type, and grade (20, 21), were extracted (**Table S1**). Next, we narrowed the target research objects using the following including criteria: (1) T1N0M0 (FIGO Stage I) OCCC, (2) diagnosed as primary tumor, (3) with known tumor size information, and (4) with follow-up and survival time information. Patients with advanced stage disease, multiple primary cancer, missing tumor size, and follow-up information were excluded. The workflow is shown in **Figure 1**. The baseline information of included patients was evaluated using the “tableone” R package and is shown in **Table 1**.

**Table 1:** Basic clinicopathological characteristics of 1,000 included stage I ovarian clear cell carcinoma.

Characteristics	Total (N=1000)
Age at diagnosis [mean (SD)]	54.85 (11.05)
Race (%)	
American Indian/Alaska Native	11 ( 1.1)
Asian or Pacific Islander	196 (19.6)
Black	27 ( 2.7)
Unknown	3 ( 0.3)
White	763 (76.3)
T_sub_class (%)	
T1	7 ( 0.7)
T1a	552 (55.2)
T1b	21 ( 2.1)
T1c	420 (42.0)
Grade* (%)	
I	15 ( 1.5)
II	83 ( 8.3)
III	297 (29.7)
IV	202 (20.2)
Unknown	403 (40.3)
tumor_size [mm, mean (SD)]	112.38 (74.75)

*The defination of tumor grade is according to coding manual of SEER database.(https://seer.cancer.gov/archive/manuals/2021/SPCSM_2021_MainDoc.pdf).

### Cutoff Point Definition

To determine the best cutoff point for tumor size, we used the X-Tile software (22), according to the manufacturer’s guidelines. Briefly, the cutoff point was defined as the risk score that generated the largest value of *χ* ^2^ in the Mantel Cox test.

### Survival Analysis

We conducted survival analysis using the “survival” package in R. Kaplan–Meier curves were used to evaluate overall survival between different groups. Log-rank *p*-values were calculated with hazard ratios and 95% confidence interval (CI) using the cox.ph function.

### Balancing the Possible Confounding Factors

To balance these possible confounding factors, namely, age at diagnosis, T stage, and degree of tumor differentiation. we used three different methods: stratification analysis, propensity score matching, and inverse probability weighting (IPW) analysis. Propensity score matching analysis was performed using the R “MatchIt” package with the parameters “method = ‘nearest’, ratio = 1”. Moreover, we further performed IPW analysis (23) using the RISCA (24) package in R.

## Results

### Basic Clinicopathological Characteristics of Stage I OCCC Patients

A total of 3,035 OCCC cancer patients were identified using ICD-O-3 codes 8310/3, 8312/3, and 8313/3. Then, we screened the candidates by using the including and excluding criteria as described in the *Method and Materials* section. A total of 1,000 stage I OCCC cases were included in the subsequent analyses. Among them, 19.6% (196/1,000) of OCCC cases were diagnosed before 45 years old (**Figure S1**). Mean tumor size of included patients is 11.24 cm (range from 0.11 to 98.9 cm, **Figure S2**). The patients’ basic clinicopathological characteristics are listed in **Table 1**.

### Tumor Size Is a Promising Prognostic Factor in Stage I OCCC Patients

Of these 1,000 included patients, median follow-up was 106 months (range from 1 to 366 months; 95% CI: 98–114). The 5-year overall survival rate for all included patients was 84.0% [standard error (SE) 1.26%]. The 5-year overall survival rate was 87.3% (SE 1.52%) for patients with stage IA disease and 78.6% (SE 2.22%) for patients with stage IC disease. Older age (age > 45 years old) was associated with inferior overall survival (**Figure S3**, *p* = 0.0099). Younger patients (age ≤ 45 years old) and the older age group had a 5-year overall survival rate of 85.4% (SE 2.71%) and 83.4% (SE 1.43%), respectively.

On univariate analysis (**Table 2**), age of diagnosis (HR 0.608, 95% CI: 0.4152–0.8910, *p* = 0.0107), stage IC (HR 1.5783, 95% CI: 1.2025–2.0716, *p* = 0.001), and tumor size (HR 1.0020, 95% CI: 1.0008–1.0033, *p* = 0.0008) were associated with prognosis. However, race, tumor differentiated degree, and stage IB were not associated with prognosis. In the multivariate analysis, age of diagnosis (HR 0.6425, 95% CI: 0.4383–0.9419, *p* = 0.0234), stage IC (HR 1.5086, 95% CI: 1.1488–1.9809, *p* = 0.0031), and tumor size (HR1.0021, 95% CI: 1.0008–1.0033, *p* = 0.0012) were independently associated with overall survival.

**Table 2:** Univariate and multivariate analysis of the included 1,000 OCCC patients.

	Univariate analysis				Multivariate analysis			
	HR	95% CI		P value	HR	95% CI		P value
		Lower	Upper			Lower	Upper	
Age_of_diagnosis								
>45	Reference				Reference			
<=45	0.608	0.4152	0.891	0.0107	0.6425	0.4383	0.9419	0.0234
Grade								
I	Reference							
II	0.738	0.278	1.9587	0.542				
III	0.8411	0.3381	2.0921	0.71				
IV	0.6776	0.2639	1.7394	0.418				
Unkonwn	0.8235	0.3345	2.0277	0.673				
Race								
American Indian/Alaska Native	Reference							
Asian or Pacific Islander	1.6522	0.2264	12.0532	0.62				
Black	2.9722	0.3576	24.7	0.313				
White	2.0221	0.2831	14.4418	0.483				
Unknown	1.61E-06	0	Inf	0.994				
Sub_stage								
IA	Reference				Reference			
IB	1.5817	0.6447	3.8809	0.3167	1.4434	0.5877	3.5451	0.4234
IC	1.5783	1.2025	2.0716	0.001	1.5086	1.1488	1.9809	0.0031
INOS	3.62E-07	0	Inf	0.9921	2.93E-07	0	Inf	0.9922
Tumor_size	1.002	1.0008	1.0033	0.0008	1.0021	1.0008	1.0033	0.0012

**Figure 2:**
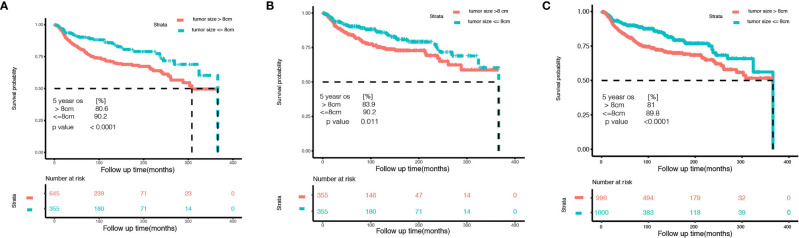
Kaplan–Meier curves of the small tumor size group (tumor size ≤ 8 cm) and the larger group (tumor size > 8 cm) of stage I ovarian clear cell carcinoma. **(A)** Before controlling potential confounding factors. **(B)** After 1:1 propensity score matching. **(C)** After inverse probability weighting correction.

Among 1,000 stage I OCCC patients, we identified 8 cm as the best cutoff point criteria according to the X-Tile software (**Table S2**). This cutoff was also observed in the T1a and T1c subgroup analysis (**Tables S3**, **S4**). Thus, we choose 8 cm as the cutoff point, and divide the patients into two subgroups. **Table 3** lists the baseline information between the two groups before and after the potential confounding factors were balanced. Then, we compared the overall survival between the two groups. As shown in **Figure 2**, OCCC patients with tumors ≤ 8 cm enjoy a significantly better prognosis than patients with a tumor > 8 cm (HR 0.5608, 95% CI: 0.4126–0.7622, *p* = 0.0002). The 5-year overall survival rate of patients with tumor size > 8 cm was 80.6%, while that for patients with tumors ≤ 8 cm was 90.2% (*p* < 0.0001). After PSM and IPW, we also found that patients with tumors ≤ 8 cm enjoyed a significantly better prognosis (**Figure 2**). We further performed subgroup analysis in stage 1a and stage 1c OCCC patients, respectively. As shown in **Figures S4**, **S5**, 8 cm could divide patients into two significantly different prognosis groups in both stage Ia and stage Ic OCCC patients.

**Table 3:** Baseline information of all T1 OCCC patients between the two groups before and after balancing the confounding factor.

Characteristics	Unmatched			Propensity Score Match			Inverse Probability Weighting		
	tumor size >8cm	tumor size <=8cm	p value	tumor size >8cm	tumor size <=8cm	p value	tumor size >8cm	tumor size <=8cm	p value
	n=645	n=355		n=355	n=355		n=996.4	n=1000	
Age (mean (SD))	55.58 (10.89)	53.52 (11.24)	0.005	53.88 (10.95)	53.52 (11.24)	0.665	54.93 (11.63)	54.86 (10.96)	0.93
Race (%)			0.15			0.933			0.839
American Indian/Alaska Native	9 (1.4)	2 (0.6)		1 (0.3)	2 (0.6)		9.6 ( 1.0)	11.0 ( 1.1)	
Asian or Pacific Islander	125 (19.4)	71 (20.0)		70 (19.7)	71 (20.0)		197.1 (19.8)	196.7 (19.7)	
Black	22 (3.4)	5 (1.4)		6 (1.7)	5 (1.4)		25.8 ( 2.6)	26.9 ( 2.7)	
Unknown	3 (0.5)	0 (0.0)					0.0 ( 0.0)	3.0 ( 0.3)	
White	486 (75.3)	277 (78.0)		278 (78.3)	277 (78.0)		763.9 (76.7)	762.4 (76.2)	
T (%)			0.103			0.702			0.986
T1	6 (0.9)	1 (0.3)		0 (0.0)	1 (0.3)		7.5 ( 0.8)	7.0 ( 0.7)	
T1a	340 (52.7)	212 (59.7)		215 (60.6)	212 (59.7)		554.9 (55.7)	553.0 (55.3)	
T1b	16 (2.5)	5 (1.4)		7 (2.0)	5 (1.4)		17.1 ( 1.7)	20.6 ( 2.1)	
T1c	283 (43.9)	137 (38.6)		133 (37.5)	137 (38.6)		416.8 (41.8)	419.4 (41.9)	
Grade (%)			0.061			0.969			1
I	10 (1.6)	5 (1.4)		4 (1.1)	5 (1.4)		15.5 ( 1.6)	15.1 ( 1.5)	
II	47 (7.3)	36 (10.1)		32 (9.0)	36 (10.1)		80.1 ( 8.0)	81.9 ( 8.2)	
III	209 (32.4)	88 (24.8)		90 (25.4)	88 (24.8)		296.3 (29.7)	297.2 (29.7)	
IV	133 (20.6)	69 (19.4)		66 (18.6)	69 (19.4)		201.8 (20.2)	202.1 (20.2)	
Unknown	246 (38.1)	157 (44.2)		163 (45.9)	157 (44.2)		402.8 (40.4)	403.7 (40.4)	

## Discussion

In this retrospective cohort study, we demonstrated the effect of tumor size on the prognosis of stage I OCCC and found that 8 cm was a promising cutoff point for tumor size. We used the cutoff to divide stage I OCCC patients into two groups and found a significant difference in prognosis.

However, limited to the clinicopathologic information we can obtain from the SEER database, we failed to obtain information on surgery approach (such as minimally invasive surgery or traditional transabdominal operation), adjuvant therapy, and disease-free survival time. Thus, in the current study, we can hardly establish the relationship between tumor size and disease-free survival. Even though we performed stratification analysis, propensity score matching, and IPW analysis to balance the potential confounding factors, such unavailable information may serve as potential confounding factors in the current study.

The majority of OCCC cancer patients are diagnosed at an early stage. However, stage I OCCC patients have a highly heterogeneous prognosis. Oseledchyk et al. (25) reported that stage IA/B patients have a significantly better prognosis than stage IC patients. However, another single-center study (26) from Japan showed no significant difference in prognosis between stage IA and rupture-only stage IC disease. Many prognostic factors in CCC have been investigated, while tumor size was seldom mentioned (8, 27, 28). Chan et al. (3) analyzed 1,411 patients with clear cell ovarian cancers and identified disease stage, age at diagnosis, and tumor grade as predictors of cancer-specific survival. Furthermore, Matsuo et al. (29) reported that lympho-vascular space invasion was an independent predictor of prognosis of stage I OCCC.

Tumor size is considered as an independent prognostic factor of clear renal cell carcinoma, and is significantly associated with lympho-vascular space invasion (30, 31). For some gynecological malignancies, the tumor size is incorporated into the FIGO stage, such as cervical cancer (32), vulvar tumor (33), and uterine sarcoma (34). In ovarian cancer, it has been widely accepted that residual tumor size after primary cytoreductive surgery is one of most important clinical predictors of patients’ prognosis (35, 36). However, the prognostic value of primary tumor size in early-stage ovarian cancer has not been well explored. Our previous single-center retrospective study (8, 37) has taken tumor size into consideration. Limited to the small population size and analysis method, we failed to find the prognosis value of primary tumor size of OCCC progression-free survival and overall survival. In this manuscript, we found that tumor size is an independent prognostic factor of stage I OCCC. A recent study reported that tumor size was independently associated with lympho-vascular space invasion in stage I OCCC (29). The odds ratio of the tumor size ≥ 15 cm group was 5.11 (95% CI: 1.37–19.1, *p* = 0.015) when compared with the tumor size < 10 cm group (29). This finding may help explain why the large tumor size group is associated with worse prognosis. In the report, the authors divided patients into three groups (<10 cm, 10–15 cm, and >15 cm), but they found that tumor size group was not significantly associated with disease-free survival and overall survival (29). Further studies will be conducted to investigate the relationship between tumor size and disease-free survival in stage I OCCC patients.

Another notable matter is fertility preservation of unexpected OCCC during ovarian cyst excision. Many OCCC patients are diagnosed at a younger age and are eager to get pregnant (4). In the current study, we found that 19.6% (196/1,000) of OCCC cases were diagnosed before 45 years old, and they enjoyed a significantly better prognosis (**Figure S3**). Several studies have reported the safety of fertility-sparing surgery among young early-stage OCCC patients. The single-center retrospective study reported by Park et al. (38) showed that there was no significant difference in 5-year disease-free survival and 5-year overall survival between the fertility-preservation group and the radical survival group. A recent systematic review also confirmed that fertility-sparing surgery is safe and feasible in low-risk early-stage OCCC patients (39). These observations suggest that further stratification management should be considered for stage I OCCC, and a fertility-preservation strategy can be considered in certain low-risk stage I OCCC patients. Our findings may provide a stratification management strategy for stage I OCCC patients. However, due to the limited high-quality evidence available, in the current version of the National Comprehensive Cancer Network (NCCN) guidelines (https://www.nccn.org/guidelines/guidelines-detail?category=1&id=1453), patients with OCCC are not recommended to undergo fertility-preserving treatment strategies, even for stage IA to IC OCCC patients. An ongoing prospective clinical research (study number: JCOG-1203) conducted by the Japan Clinical Oncology Group (JCOG) will provide high-quality evidence of the safety of the fertility-sparing strategy in stage I OCCC patients (https://jrct.niph.go.jp/latest-detail/jRCTs031180178).

## Conclusion

Tumor size is an independent prognostic factor for stage I OCCC. Additionally, 8 cm is a promising cutoff point for tumor size for risk stratification. However, the use of tumor size in the stratification management of stage I OCCC patients warrants further investigation.

## Data Availability Statement

The datasets presented in this study can be found in online repositories. The names of the repository/repositories and accession number(s) can be found in the article/**Supplementary Material**.

## Author Contributions

LW, HS, and HZ designed the study. Data collection: LW and SS. Data analysis and interpretation: LW, HS, and HZ. Manuscript writing and figure preparation: LW and SS. All authors contributed to the article and approved the submitted version.

## Funding

This study was supported by the National Natural Science Foundation of China (Grant No. 81902620).

## Conflict of Interest

The authors declare that the research was conducted in the absence of any commercial or financial relationships that could be construed as a potential conflict of interest

## Publisher’s Note

All claims expressed in this article are solely those of the authors and do not necessarily represent those of their affiliated organizations, or those of the publisher, the editors and the reviewers. Any product that may be evaluated in this article, or claim that may be made by its manufacturer, is not guaranteed or endorsed by the publisher.

## Supplementary Material

Supplementary Figure 1 Age distribution of included stage I ovarian clear cell carcinoma. *x*-axis: age of diagnosis (years old), *y*-axis: number of cases.

Supplementary Figure 2 Tumor size distribution of included stage I ovarian clear cell carcinoma. *x*-axis: tumor size (in millimeters), *y*-axis: number of cases.

Supplementary Figure 3 Kaplan–Meier curves of young (age <= 45 years old) and old (age > 45 years old) group stage I ovarian clear cell carcinoma.

Supplementary Figure 4 Kaplan–Meier curves of the small tumor size group (tumor size ≤ 8 cm) and the larger group (tumor size > 8 cm) of stage Ia ovarian clear cell carcinoma. **(A)** Before controlling potential confounding factors. **(B)** After 1:1 propensity score matching. **(C)** After inverse probability weighting correction.

Supplementary Figure 5 Kaplan–Meier curves of the small tumor size group (tumor size ≤ 8 cm) and the larger group (tumor size > 8 cm) of stage Ic ovarian clear cell carcinoma. **(A)** Before controlling potential confounding factors. **(B)** After 1:1 propensity score matching. **(C)** After inverse probability weighting correction.

Supplementary Table 1 Detailed clinical pathological information of the included 1,000 OCCC patients.

Supplementary Table 2 Cutoff value screen of 1,000 stage I OCCC tumor size by X-tile software.

Supplementary Table 3 Cutoff value screen of stage Ia OCCC tumor size by X-tile software.

Supplementary Table 4 Cutoff value screen of stage Ic OCCC tumor size by X-tile software.

## References

[1] Oda K Hamanishi J Matsuo K Hasegawa K. Genomics to Immunotherapy of Ovarian Clear Cell Carcinoma: Unique Opportunities for Management. Gynecologic Oncol (2018) 151(2):381–9. doi: 10.1016/j.ygyno.2018.09.001 PMC7526052 30217369

[2] Khalique S Lord CJ Banerjee S Natrajan R. Translational Genomics of Ovarian Clear Cell Carcinoma. Semin Cancer Biol (2020) 61:121–31. doi: 10.1016/j.semcancer.2019.10.025 31698086

[3] Chan JK Teoh D Hu JM Shin JY Osann K Kapp DS. Do Clear Cell Ovarian Carcinomas Have Poorer Prognosis Compared to Other Epithelial Cell Types? A Study of 1411 Clear Cell Ovarian Cancers. Gynecologic Oncol (2008) 109(3):370–6. doi: 10.1016/j.ygyno.2008.02.006 18395777

[4] So KA Hong SR Kim NR Yang EJ Shim SH Lee SJ. Association Between Atypical Endometriosis and Ovarian Malignancies in the Real World. J Ovarian Res (2021) 14(1):110. doi: 10.1186/s13048-021-00865-2 34454550 PMC8403438

[5] Gallotta V Cicero C Conte C Vizzielli G Petrillo M Fagotti A. Robotic Versus Laparoscopic Staging for Early Ovarian Cancer: A Case-Matched Control Study. J Minim Invasive Gynecol (2017) 24(2):293–8. doi: 10.1016/j.jmig.2016.11.004 27856387

[6] Gallotta V Jeong SY Conte C Trozzi R Cappuccio S Moroni R. Minimally Invasive Surgical Staging for Early Stage Ovarian Cancer: A Long-Term Follow Up. Eur J Surg Oncol (2021) 47(7):1698–704. doi: 10.1016/j.ejso.2021.01.033 33573854

[7] Gallotta V Petrillo M Conte C Vizzielli G Fagotti A Ferrandina G. Laparoscopic Versus Laparotomic Surgical Staging for Early-Stage Ovarian Cancer: A Case-Control Study. J Minim Invasive Gynecol (2016) 23(5):769–74. doi: 10.1016/j.jmig.2016.03.006 26995493

[8] Zhao T Shao Y Liu Y Wang X Guan L Lu Y. Endometriosis Does Not Confer Improved Prognosis in Ovarian Clear Cell Carcinoma: A Retrospective Study at a Single Institute. J Ovarian Res (2018) 11(1):53. doi: 10.1186/s13048-018-0425-9 29941051 PMC6019519

[9] Mackenzie R Talhouk A Eshragh S Lau S Cheung D Chow C. Morphologic and Molecular Characteristics of Mixed Epithelial Ovarian Cancers. Am J Surg Pathol (2015) 39(11):1548–57. doi: 10.1097/PAS.0000000000000476 PMC4604016 26099008

[10] Zhou L Yao L Dai L Zhu H Ye X Wang S. Ovarian Endometrioid Carcinoma and Clear Cell Carcinoma: A 21-Year Retrospective Study. J Ovarian Res (2021) 14(1):63. doi: 10.1186/s13048-021-00804-1 33941230 PMC8094516

[11] Lee YY Kim TJ Kim MJ Kim HJ Song T Kim MK. Prognosis of Ovarian Clear Cell Carcinoma Compared to Other Histological Subtypes: A Meta-Analysis. Gynecologic Oncol (2011) 122(3):541–7. doi: 10.1016/j.ygyno.2011.05.009 21640372

[12] Liu H Xu Y Ji J Dong R Qiu H Dai X. Prognosis of Ovarian Clear Cell Cancer Compared With Other Epithelial Cancer Types: A Population-Based Analysis. Oncol Lett (2020) 19(3):1947–57. doi: 10.3892/ol.2020.11252 PMC7038925 32194689

[13] Gadducci A Multinu F Cosio S Carinelli S Ghioni M Aletti GD. Clear Cell Carcinoma of the Ovary: Epidemiology, Pathological and Biological Features, Treatment Options and Clinical Outcomes. Gynecologic Oncol (2021) 162(3):741–50. doi: 10.1016/j.ygyno.2021.06.033 34247767

[14] Öcal O Ingrisch M Ümütlü MR Peynircioglu B Loewe C van Delden O. Prognostic Value of Baseline Imaging and Clinical Features in Patients With Advanced Hepatocellular Carcinoma. Br J Cancer (2021) 126(2):211–8. doi: 10.1038/s41416-021-01577-6 PMC8770679 34686780

[15] Wang DF Zhang GN Peng CR Shi Y Shi XW. Analysis of Factors Related to the Prognostic Benefit of Neoadjuvant Chemotherapy Followed by Interval Debulking Surgery in Patients With Advanced Ovarian Cancer. Zhonghua Fu Chan Ke Za Zhi (2021) 56(6):385–92. doi: 10.3760/cma.j.cn112141-20201207-00871 34154313

[16] Ling S Hu Z Yang Z Yang F Li Y Lin P. Extremely High Genetic Diversity in a Single Tumor Points to Prevalence of non-Darwinian Cell Evolution. Proc Natl Acad Sci USA (2015) 112(47):E6496–6505. doi: 10.1073/pnas.1519556112 PMC4664355 26561581

[17] Chien J Neums L Powell A Torres M Kalli KR Multinu F. Genetic Evidence for Early Peritoneal Spreading in Pelvic High-Grade Serous Cancer. Front Oncol (2018) 8:58. doi: 10.3389/fonc.2018.00058 29594039 PMC5858520

[18] Schwarz RF Ng CK Cooke SL Newman S Temple J Piskorz AM. Spatial and Temporal Heterogeneity in High-Grade Serous Ovarian Cancer: A Phylogenetic Analysis. PloS Med (2015) 12(2):e1001789. doi: 10.1371/journal.pmed.1001789 25710373 PMC4339382

[19] Sharma VP Tang B Wang Y Duran CL Karagiannis GS Xue EA. Live Tumor Imaging Shows Macrophage Induction and TMEM-Mediated Enrichment of Cancer Stem Cells During Metastatic Dissemination. Nat Commun (2021) 12(1):7300. doi: 10.1038/s41467-021-27308-2 34911937 PMC8674234

[20] Ryu SY Park SI Nam BH Kim I Yoo CW Nam JH. Prognostic Significance of Histological Grade in Clear-Cell Carcinoma of the Ovary: A Retrospective Study of Korean Gynecologic Oncology Group. Ann Oncol (2009) 20(6):1032–6. doi: 10.1093/annonc/mdn764 19193704

[21] Lin LH Zamuco RD Shukla PS. Intratumoral Budding is Associated With Poor Clinical Outcome in Early-Stage Clear Cell Carcinoma of Ovary. Histopathology (2021) 79(6):1018–29. doi: 10.1111/his.14459 34292622

[22] Camp RL Dolled-Filhart M Rimm DL. X-Tile: A New Bio-Informatics Tool for Biomarker Assessment and Outcome-Based Cut-Point Optimization. Clin Cancer Res (2004) 10(21):7252–9. doi: 10.1158/1078-0432.CCR-04-0713 15534099

[23] Narduzzi S Golini MN Porta D Stafoggia M Forastiere F. Inverse Probability Weighting (IPW) for Evaluating and "Correcting" Selection Bias. Epidemiol Prev (2014) 38(5):335–41. 25387748

[24] Chatton A Le Borgne F Leyrat C Gillaizeau F Rousseau C Barbin L. G-Computation, Propensity Score-Based Methods, and Targeted Maximum Likelihood Estimator for Causal Inference With Different Covariates Sets: A Comparative Simulation Study. Sci Rep (2020) 10(1):9219. doi: 10.1038/s41598-020-65917-x 32514028 PMC7280276

[25] Oseledchyk A Leitao MM Jr. Konner J O'Cearbhaill RE Zamarin D Sonoda Y. Adjuvant Chemotherapy in Patients With Stage I Endometrioid or Clear Cell Ovarian Cancer in the Platinum Era: A Surveillance, Epidemiology, and End Results Cohort Study, 2000-2013. Ann Oncol (2017) 28(12):2985–93. doi: 10.1093/annonc/mdx525 PMC5834056 28950307

[26] Shu CA Zhou Q Jotwani AR Iasonos A Leitao MM Jr. Konner JA. Ovarian Clear Cell Carcinoma, Outcomes by Stage: The MSK Experience. Gynecologic Oncol (2015) 139(2):236–41. doi: 10.1016/j.ygyno.2015.09.016 PMC4632203 26404183

[27] Hogen L Brar H Covens A Bassiouny D Bernardini MQ Gien LT. Is Adjuvant Chemotherapy Beneficial for Surgical Stage I Ovarian Clear Cell Carcinoma? Gynecologic Oncol (2017) 147(1):54–60. doi: 10.1016/j.ygyno.2017.07.128 28760368

[28] Nasioudis D Mastroyannis SA Albright BB Haggerty AF Ko EM Latif NA. Adjuvant Chemotherapy for Stage I Ovarian Clear Cell Carcinoma: Patterns of Use and Outcomes. Gynecologic Oncol (2018) 150(1):14–8. doi: 10.1016/j.ygyno.2018.04.567 29751993

[29] Matsuo K Yoshino K Hasegawa K Murakami R Ikeda Y Adachi S. Survival Outcome of Stage I Ovarian Clear Cell Carcinoma With Lympho-Vascular Space Invasion. Gynecologic Oncol (2015) 136(2):198–204. doi: 10.1016/j.ygyno.2014.12.006 25497604

[30] Tang Y Liu F Mao X Li P Mumin MA Li J. The Impact of Tumor Size on the Survival of Patients With Small Renal Masses: A Population-Based Study. Cancer Med (2022). doi: 10.1002/cam4.4595 PMC9189465 35229988

[31] Wang J Tang J Chen T Yue S Fu W Xie Z. A Web-Based Prediction Model for Overall Survival of Elderly Patients With Early Renal Cell Carcinoma: A Population-Based Study. J Transl Med (2022) 20(1):90. doi: 10.1186/s12967-022-03287-w 35164796 PMC8845298

[32] Bhatla N Aoki D Sharma DN Sankaranarayanan R. Cancer of the Cervix Uteri. Int J Gynaecol Obstet (2018) 143(Suppl 2):22–36. doi: 10.1002/ijgo.12611 30306584

[33] Rogers LJ Cuello MA. Cancer of the Vulva. Int J Gynaecol Obstet (2018) 143(Suppl 2):4–13. doi: 10.1002/ijgo.12609 30306583

[34] Amant F Mirza MR Koskas M Creutzberg CL. Cancer of the Corpus Uteri. Int J Gynaecol Obstet (2018) 143(Suppl 2):37–50. doi: 10.1002/ijgo.12612 30306580

[35] Polterauer S Vergote I Concin N Braicu I Chekerov R Mahner S. Prognostic Value of Residual Tumor Size in Patients With Epithelial Ovarian Cancer FIGO Stages IIA-IV: Analysis of the OVCAD Data. Int J gynecol Cancer Off J Int Gynecol Cancer Soc (2012) 22(3):380–5. doi: 10.1097/IGC.0b013e31823de6ae 22266934

[36] Uzan J Bonsang-Kitzis H Rossi L Rance B Bats AS Gosset M. Prognostic Impact of Initial Tumor Load and Intraperitoneal Disease Dissemination Patterns in Patients With Advanced Ovarian Cancer Undergoing Complete Cytoreductive Surgery. Eur J Surg Oncol (2019) 45(9):1619–24. doi: 10.1016/j.ejso.2019.04.011 31014987

[37] Tang H Liu Y Wang X Guan L Chen W Jiang H. Clear Cell Carcinoma of the Ovary: Clinicopathologic Features and Outcomes in a Chinese Cohort. Med (Baltimore) (2018) 97(21):e10881. doi: 10.1097/MD.0000000000010881 PMC6392688 29794794

[38] Park JY Suh DS Kim JH Kim YM Kim YT Nam JH. Outcomes of Fertility-Sparing Surgery Among Young Women With FIGO Stage I Clear Cell Carcinoma of the Ovary. Int J Gynaecol Obstet (2016) 134(1):49–52. doi: 10.1016/j.ijgo.2015.10.022 27039052

[39] Prodromidou A Theofanakis C Thomakos N Haidopoulos D Rodolakis A. Fertility Sparing Surgery for Early-Stage Clear Cell Carcinoma of the Ovary; A Systematic Review and Analysis of Obstetric Outcomes. Eur J Surg Oncol (2021) 47(6):1286–91. doi: 10.1016/j.ejso.2021.01.016 33509613

