# Supplementary figures and images for: Tumor Size Is an Independent Prognostic Factor for Stage I Ovarian Clear Cell Carcinoma: A Large Retrospective Cohort Study of 1,000 Patients
Source: Front Oncol. 2022 May 16;12:862944. doi: 10.3389/fonc.2022.862944 (PMC9149085; doi:10.3389/fonc.2022.862944)

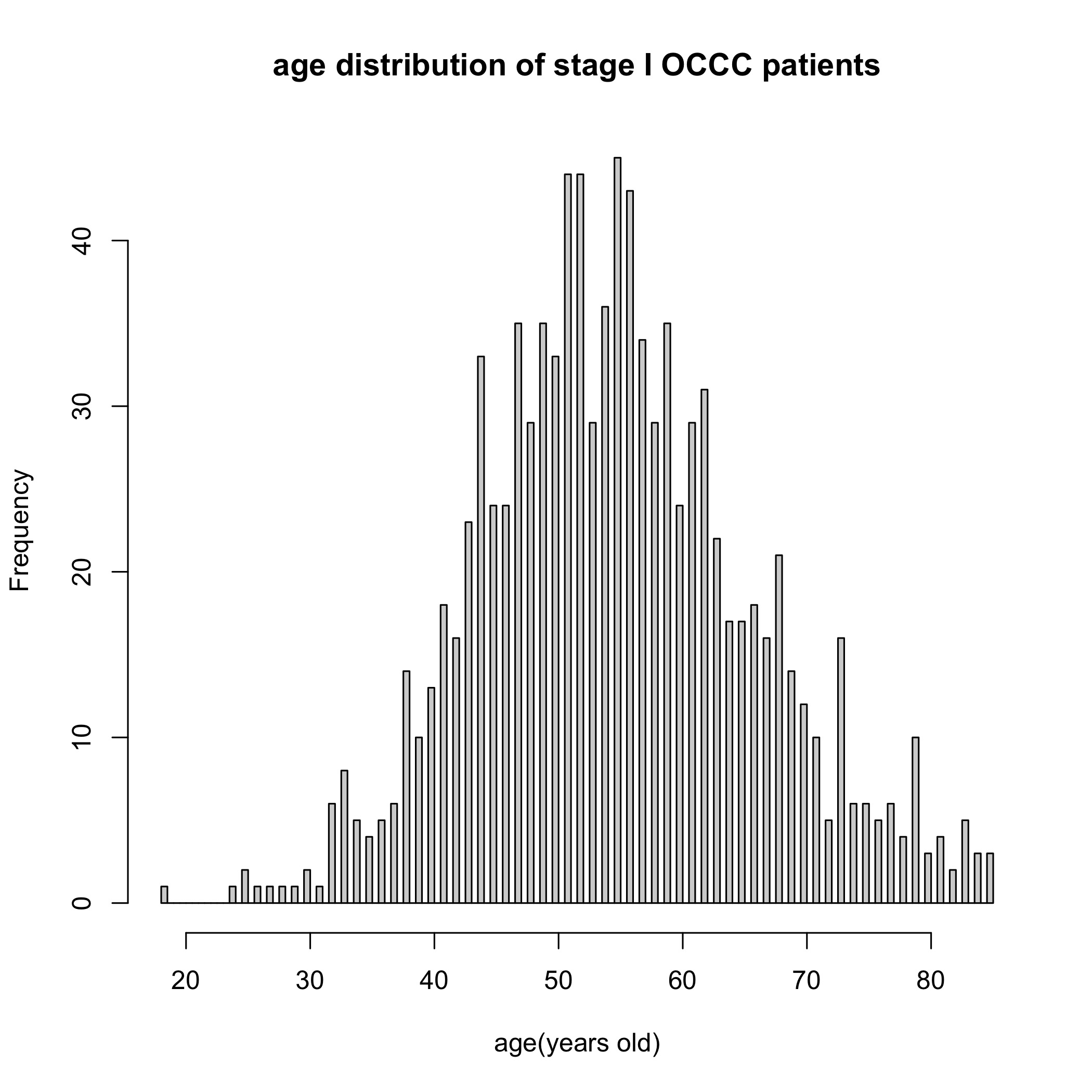

Supplement: Supplementary Figure 1 — Age distribution of included stage I ovarian clear cell carcinoma. x-axis: age of diagnosis (years old), y-axis: number of cases. [file Image_1.jpeg]

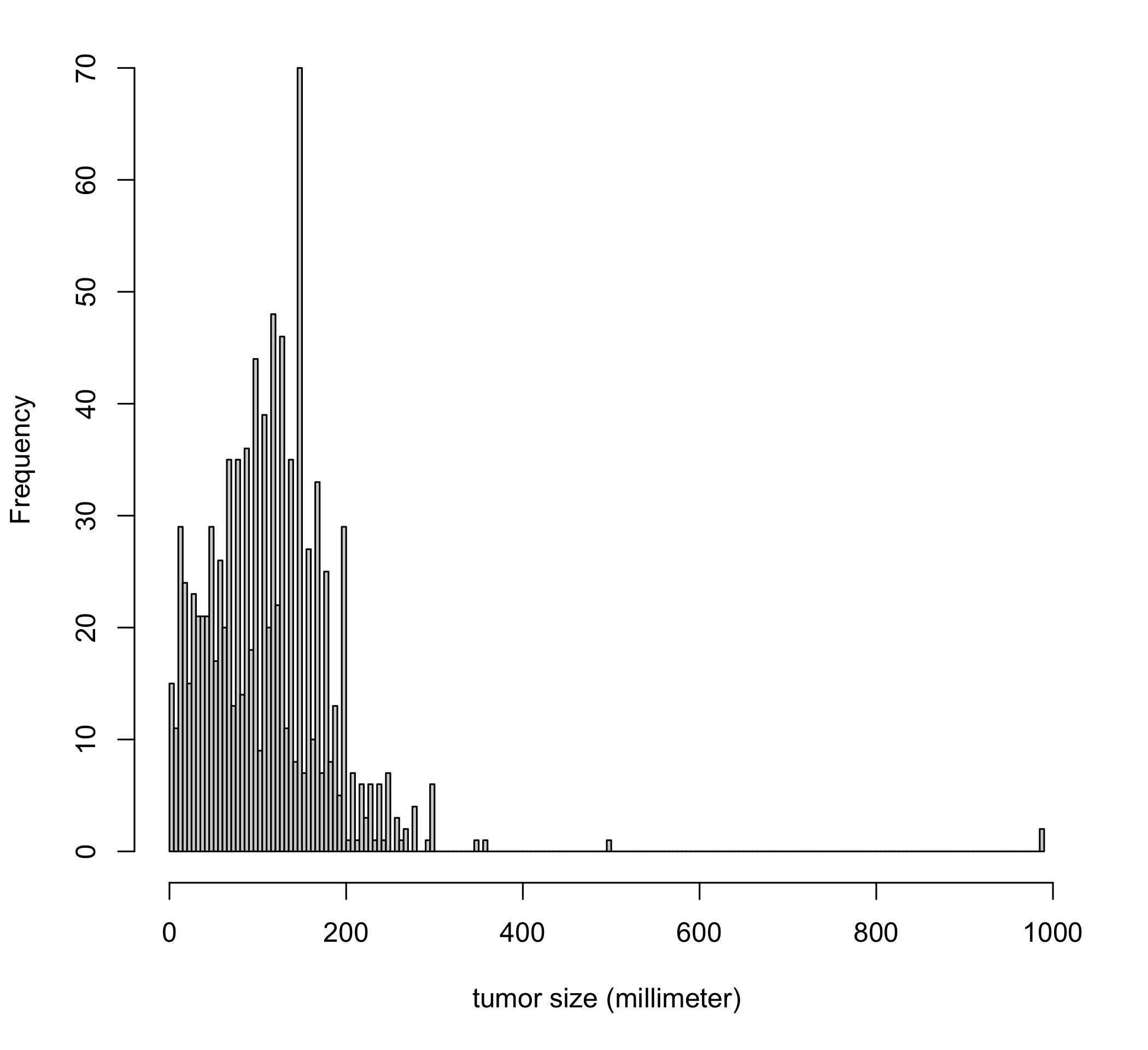

Supplement: Supplementary Figure 2 — Tumor size distribution of included stage I ovarian clear cell carcinoma. x-axis: tumor size (in millimeters), y-axis: number of cases. [file Image_2.jpg]

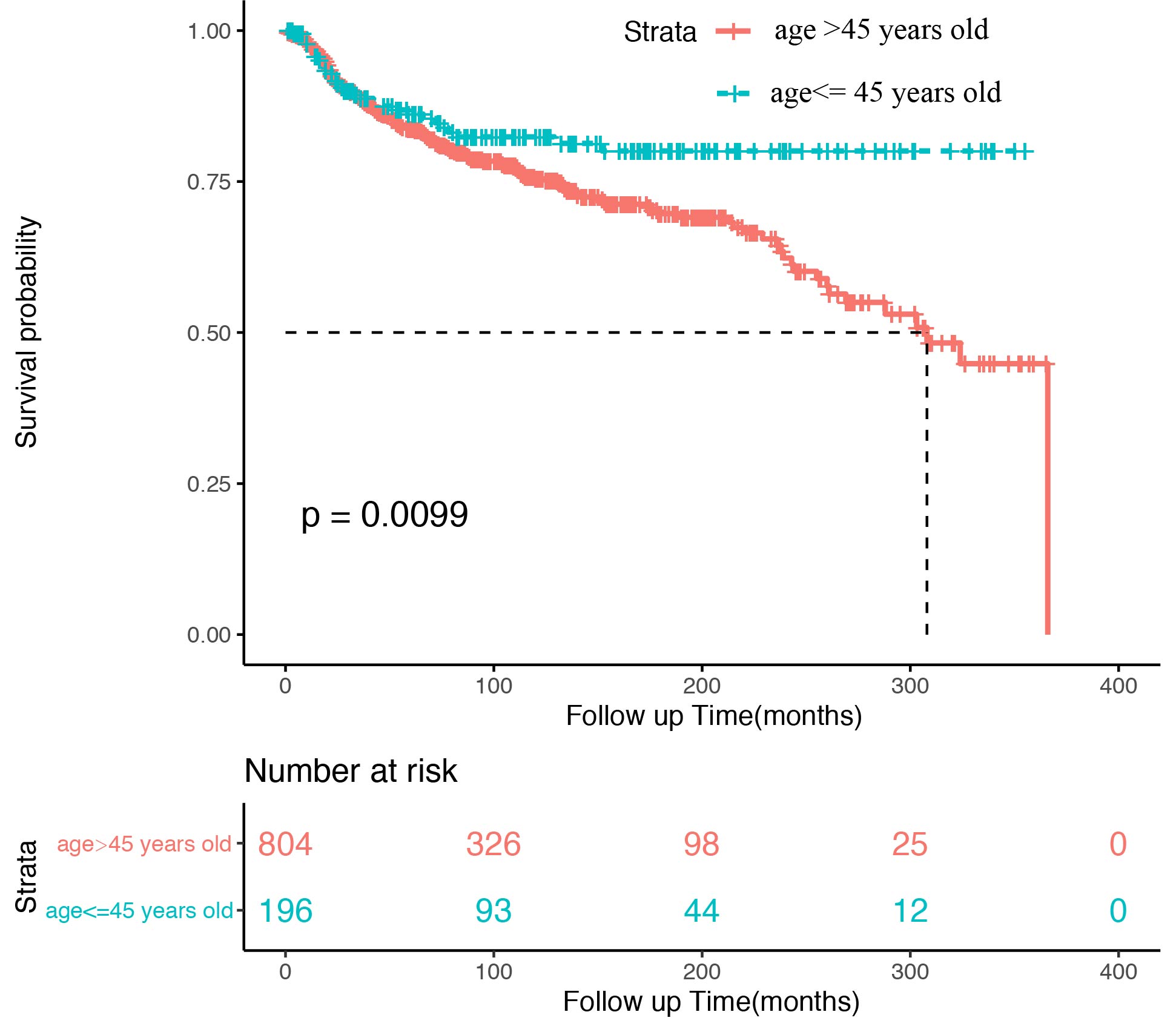

Supplement: Supplementary Figure 3 — Kaplan–Meier curves of young (age <= 45 years old) and old (age > 45 years old) group stage I ovarian clear cell carcinoma. [file Image_3.jpeg]

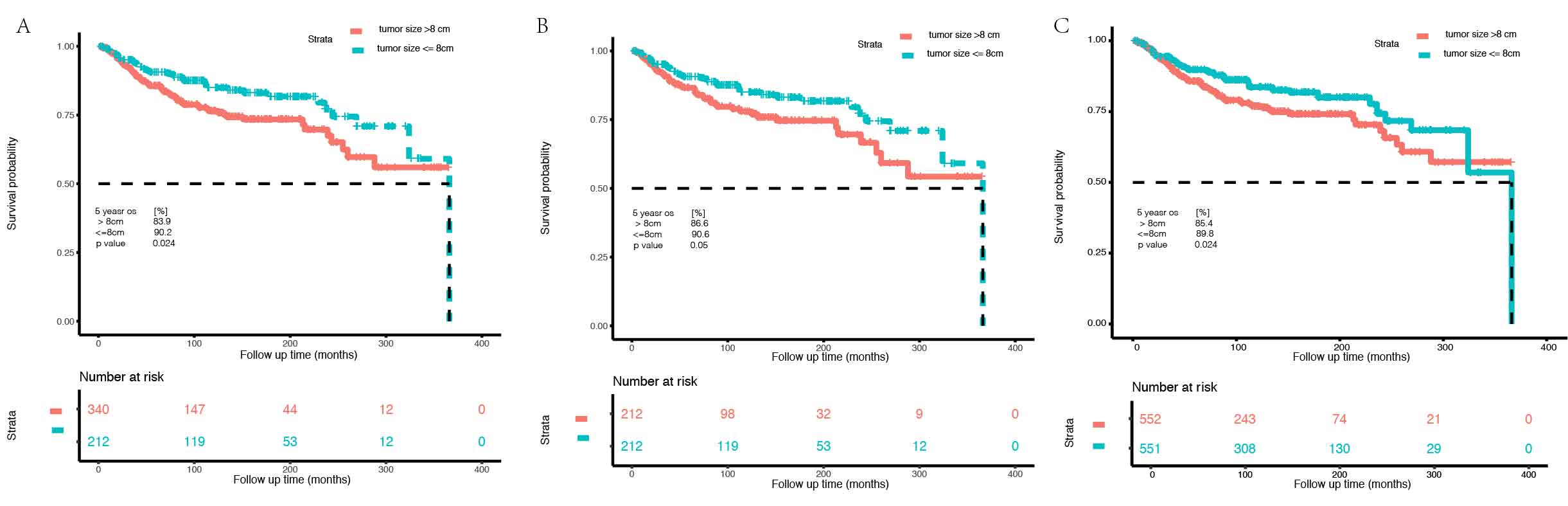

Supplement: Supplementary Figure 4 — Kaplan–Meier curves of the small tumor size group (tumor size ≤ 8 cm) and the larger group (tumor size > 8 cm) of stage Ia ovarian clear cell carcinoma. (A) Before controlling potential confounding factors. (B) After 1:1 propensity score matching. (C) After inverse probability weighting correction. [file Image_4.jpeg]

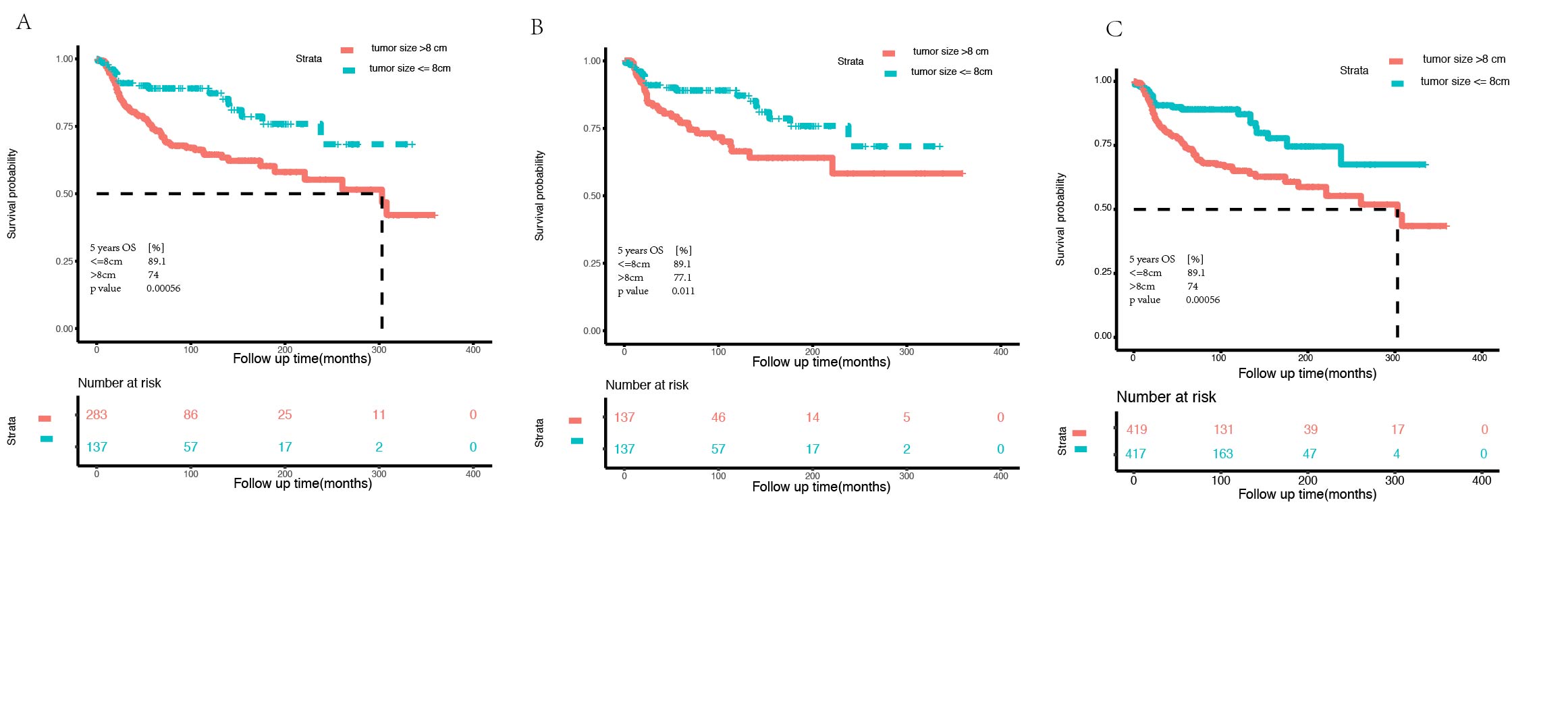

Supplement: Supplementary Figure 5 — Kaplan–Meier curves of the small tumor size group (tumor size ≤ 8 cm) and the larger group (tumor size > 8 cm) of stage Ic ovarian clear cell carcinoma. (A) Before controlling potential confounding factors. (B) After 1:1 propensity score matching. (C) After inverse probability weighting correction. [file Image_5.jpeg]
